# Gastrointestinal Hemorrhage in CREST Syndrome: A Case Report

**DOI:** 10.7759/cureus.85978

**Published:** 2025-06-14

**Authors:** Jayashree Ravikumar, Thalvayapati Sai Prudhvi

**Affiliations:** 1 Internal Medicine, Sri Varadhar Consultation Clinic, Chennai, IND; 2 Internal Medicine, Kilpauk Medical College, Chennai, IND

**Keywords:** calcinosis, crest syndrome, gastrointestinal bleed, gastrointestinal hemorrhage, limited scleroderma, scleroderma

## Abstract

Systemic scleroderma is an autoimmune disorder caused by microvascular dysfunction, excessive collagen deposition, and progressive fibrosis affecting the skin and other organs. CREST syndrome, also known as limited scleroderma, is an acronym for calcinosis, Raynaud’s phenomenon, esophageal dysfunction, sclerodactyly, and telangiectasias. Among patients with systemic sclerosis (SSc), anticentromere antibodies are typically associated with the CREST syndrome variant. These antibodies are considered disease-specific and are known to correlate with the extent of disease progression. The pathogenesis of SSc is complex, with unusual presentations increasingly seen among patients. Gastrointestinal (GI) hemorrhage can be observed in CREST syndrome as an uncommon but serious complication. Endoscopic interventions are warranted in such patients to evaluate and manage bleeding. This report aims to raise awareness among physicians that CREST syndrome can present with GI hemorrhage, with skin signs such as calcinosis and sclerodactyly. In our case, H2 blockers and proton pump inhibitors were administered as part of the management plan to reduce acid-related mucosal injury and mitigate GI bleeding. Due to chronic mucosal hemorrhage, anemia was noted and corrected with parenteral iron therapy. The prognosis of CREST syndrome is relatively good when compared to the diffuse variant. Further research is needed to understand the pathophysiology of CREST syndrome, reliable biomarkers for early diagnosis, and targeted preventive treatment for this condition. Sensitizing clinicians regarding the changing disease pattern will help in prompt diagnosis.

## Introduction

CREST syndrome is a limited form of systemic sclerosis (SSc), an autoimmune disease characterized by vascular abnormalities, fibrosis, and specific autoantibodies. The acronym "CREST" refers to five clinical features: calcinosis, Raynaud’s phenomenon, esophageal dysmotility, sclerodactyly, and telangiectasia. Among these, esophageal involvement is common, yet overt gastrointestinal (GI) hemorrhage is considered rare, especially in patients with the CREST subtype [1, 2]. The pathophysiology of GI involvement in SSc includes vascular dysfunction, smooth muscle atrophy, and fibrosis, which impair GI motility and mucosal integrity. Telangiectasias, dilated small blood vessels typically found in the stomach and esophagus, are recognized as potential sources of GI bleeding in these patients [3]. However, there is limited literature specifically addressing GI hemorrhage in CREST syndrome, and no standardized treatment protocols currently exist. This case report describes an unusual presentation of GI bleeding in a patient with CREST syndrome, discusses diagnostic challenges in resource-limited settings, and reviews current evidence and management approaches for this complication.

## Case presentation

A 56-year-old multiparous Indian woman presented in the outpatient department of a tertiary academic medical center located in a resource-limited region with complaints of intermittent hematemesis over the past eight months, accompanied by persistent fatigue, generalized weakness, and intermittent upper abdominal discomfort. The episodes of hematemesis were characterized by the vomiting of approximately 50-100 mL of fresh blood, often occurring in the early morning hours on an empty stomach. The patient was managed at a tertiary academic medical center in a resource-limited region of South India, where access to endoscopic facilities is constrained. No significant past surgical history was reported. She denied tobacco or alcohol use and was a homemaker. There were no known genetic conditions or relevant family medical history. She had no history of previous autoimmune or GI disorders. The medication history was unremarkable. The patient reported weight loss of about 4 kg during this period.

**Figure 1:**
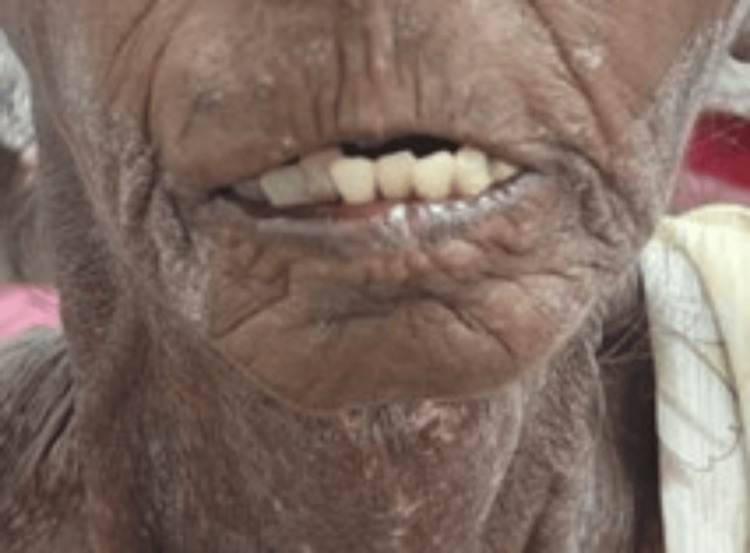
Reduced Mouth Opening (Microstomia) in our Patient With CREST Syndrome, Illustrating Cutaneous Fibrosis and Perioral Skin Tightening, Common Features in Limited Systemic Sclerosis That May Impact Oral Intake and Quality of Life CREST: Calcinosis, Raynaud’s Phenomenon, Esophageal Dysmotility, Sclerodactyly, and Telangiectasia

Physical examination revealed pallor, restricted mouth opening (Figure 1), and dry skin with visible calcinosis over the dorsal aspects of her left digits. Sclerodactyly and reduced digital mobility were noted. Cardiopulmonary and neurological examinations were unremarkable.

Laboratory investigations (Table 1) showed microcytic hypochromic anemia with a hemoglobin level of 8.8 g/dL, red blood cell count of 4 million/cu mm, and other indices suggestive of chronic iron-deficiency anemia. Antinuclear antibody was positive with a centromere pattern (1:640 titer), and anticentromere antibody levels were significantly elevated (Table 2). Ceruloplasmin and alpha-1 antitrypsin were included to rule out alternative causes of chronic liver disease and systemic symptoms, such as Wilson’s disease and alpha-1 antitrypsin deficiency, which may present with overlapping clinical features and anemia. Their normal levels helped support an autoimmune etiology rather than a metabolic or hereditary disorder.

**Table 1:** Laboratory Results of Complete Hemogram in Our Patient With CREST Syndrome CREST: Calcinosis, Raynaud’s Phenomenon, Esophageal Dysmotility, Sclerodactyly, and Telangiectasia

Laboratory Test (Unit)	Results	Reference Value
Erythrocyte (RBC) Count, mill/cu mm	4	3.80-4.80
Hemoglobin (Hb), g/dL	8.8	12-15
Packed Cell Volume, %	30	36.0-46.0
Mean Corpuscular Volume, fL	78	83.0-101.0
Mean Corpuscular Hb, pg	28	27.0-32.0
Mean Corpuscular Hb Concentration, g/dL	32	31-34.5
Red Cell Distribution Width, %	11	11.6-14.0
White Blood Cells, cells/ cu mm	8.8	4000-11000
Platelets, 10^9^/L	200	150–450
Peripheral Smear	Microcytic Hypochromic Anemia	

**Table 2:** Laboratory Result of Antibody Titers in Our Patient With CREST Syndrome CREST: Calcinosis, Raynaud’s Phenomenon, Esophageal Dysmotility, Sclerodactyly, and Telangiectasia

Parameter	Results	Normal Range
Antinuclear Antibody	1:640 (Centromere Pattern)	<40
Anti-smooth Muscle Antibody	<16	<20
Anticentromere Antibody(U)	>10.0	<1
Anti-Scl-70(U)	<0.5	<1
Anti-RNA Polymerase III	<7	<20
Anti-Th/To Ab	Negative	Negative
Ceruloplasmin (mg/dL)	36	18-36
Alpha-1 Antitrypsin (mg/dL)	205	83-199

The differential diagnosis for this patient’s presentation included several possibilities. Peptic ulcer disease was initially considered due to the hematemesis, but the absence of nonsteroidal anti-inflammatory drug (NSAID) use, alcohol history, or epigastric pain made this less likely. Esophageal varices were another possibility, yet there were no signs of chronic liver disease or portal hypertension. Gastric or esophageal malignancy was considered; however, the absence of significant weight loss, cachexia, or GI alarm symptoms made it less likely, though it could not be definitively excluded without endoscopic evaluation. A Mallory-Weiss tear was ruled out due to the absence of preceding retching or vomiting episodes. Ultimately, telangiectasia-associated bleeding due to CREST syndrome was the most consistent diagnosis, supported by the patient’s mucocutaneous findings, serological markers, and clinical course. Access to upper GI endoscopy was not available at our center, posing a diagnostic challenge. The diagnosis was instead supported by physical signs, laboratory findings, and autoimmune antibody profiles. Differential diagnoses were carefully considered and excluded based on history, clinical signs, and serology.

Treatment focused on acid suppression using oral ranitidine 150 mg twice daily and iron repletion with intravenous iron sucrose (200 mg, three times weekly). No invasive interventions were undertaken due to the clinical stability of the patient. Due to resource constraints, upper GI endoscopy was not performed. A working diagnosis of GI hemorrhage secondary to CREST syndrome was made based on clinical features and serological markers. The patient was treated conservatively with oral ranitidine 150 mg twice daily and parenteral iron sucrose (200 mg thrice weekly). Her clinical symptoms gradually improved during hospitalization with no recurrence of hematemesis.

The patient was followed up two weeks after discharge, with clinical reassessment showing no recurrence of hematemesis and a stable hemoglobin level of 11.2 g/dL. A second follow-up visit four weeks later confirmed continued improvement.

## Discussion

CREST syndrome is a variant of SSc characterized by limited cutaneous involvement and specific autoantibodies, most notably anticentromere antibodies. Although GI involvement is well-recognized in SSc, overt GI hemorrhage remains an uncommon presentation, particularly in CREST syndrome. Similar cases have been documented in the literature. Duchini and Sessoms described GI hemorrhage in CREST syndrome as primarily due to mucosal telangiectasias [1]. Miller et al. noted that GI bleeding can be the first manifestation of SSc, especially when telangiectasias are submucosal [2]. Nassar et al. emphasized the growing incidence of GI bleeding in CREST cases, linking it to progressive vascular damage [3]. The American College of Gastroenterology highlights various scoring systems, such as Rockall and Glasgow-Blatchford, for risk stratification and management of upper GI bleeding [4]. The management of GI hemorrhage in CREST syndrome is not standardized. Proton pump inhibitors and H2 blockers are commonly used to mitigate acid-related mucosal injury [5]. Endoscopic techniques, although not performed in our case, remain essential when available to identify bleeding sources and apply therapeutic interventions. 

GI manifestations in SSc result from vascular damage, smooth muscle atrophy, and fibrosis, leading to motility disorders, mucosal ischemia, and, less frequently, bleeding. Telangiectasias in the GI tract, especially in the stomach and esophagus, are the most frequently implicated lesions in bleeding cases. These lesions are typically identified via endoscopy and are thought to arise from longstanding vascular dysfunction and mucosal hypoxia due to microangiopathy [6,7].

However, in real-world clinical settings, particularly in resource-limited environments, endoscopy may not always be feasible. In such scenarios, physicians must rely on clinical acumen, laboratory findings, and autoimmune profiles to guide diagnosis and management. Our patient’s mucocutaneous findings (calcinosis, sclerodactyly), iron-deficiency anemia, and high-titer anticentromere antibodies supported a diagnosis of CREST syndrome-associated GI bleeding, even without endoscopic confirmation [8,9]. Recent evidence highlights that even in the absence of mucosal lesions detectable by endoscopy, CREST patients may develop upper GI hemorrhage due to subepithelial telangiectasias and mucosal fragility exacerbated by acid exposure [10]. In a retrospective study by Bruni et al., nearly 20% of patients with limited SSc reported at least one episode of GI bleeding, most of whom lacked classic erosive esophagitis on endoscopy [11]. Beyond telangiectasias, the role of delayed gastric emptying and decreased peristalsis in promoting mucosal injury and reflux-mediated damage has been documented, contributing to microbleeds that may accumulate over time [12]. Thus, conservative treatment focused on acid suppression, mucosal protection, and iron replacement remains central in such scenarios. The inclusion of non-invasive vascular imaging tools, such as capsule endoscopy or CT angiography, may assist in diagnosis in select cases [13]. Multidisciplinary care involving rheumatologists, gastroenterologists, and nutrition specialists can enhance management outcomes and reduce the recurrence of hemorrhagic events [14].

## Conclusions

GI hemorrhage in CREST syndrome is often secondary to mucosal or submucosal telangiectasias, linked to vascular dysfunction. In settings where endoscopy is unavailable, clinical acumen, autoimmune profiles, and supportive laboratory findings play a crucial role in diagnosis. This case underscores the effectiveness of conservative management when invasive diagnostics are inaccessible. This case highlights a rare yet significant complication of CREST syndrome. It emphasizes the importance of clinical recognition, serological confirmation, and the potential success of non-invasive therapeutic strategies in low-resource environments.
